# IR-YOLOv7-Tiny: A Lightweight and Robust Framework for Fabric-Defect Detection

**DOI:** 10.3390/s26041094

**Published:** 2026-02-08

**Authors:** Shirong Guo, Shuiguang Tong

**Affiliations:** School of Mechanical Engineering, Zhejiang University, Hangzhou 310058, China; cetongsg@zju.edu.cn

**Keywords:** defect detection, deep learning, downsampling, image denoising, YOLOv7

## Abstract

To tackle the challenges of missed detections, false alarms, electromagnetic noise, and constrained deployment resources in fabric-defect inspection, we propose a lightweight and interference-resilient fabric-defect detector based on the Discrete Wavelet Transform (DWT). First, a color-space channel separation filter leverages Hue–Saturation–Value (HSV) decomposition to suppress illumination and electromagnetic interference while preserving fabric structural details. Second, DWT is employed to extract directional texture features (horizontal, vertical, and diagonal) from complex woven structures. Third, the backbone of the You Only Look Once version 7 Tiny (YOLOv7-Tiny) is modified by replacing pooling with a Spatial Pyramid Dilated Convolution (SPD) block, which maintains fine-grained detail during downsampling. For upsampling, an inverted SPD block with channel concatenation is introduced to mitigate background redundancy caused by interpolation. Experimental results on the TILDA and DAGM datasets show that the proposed IR-YOLOv7-Tiny achieves mAP@0.5 of 96.8% and 98.8%, respectively, with only 3.5 M parameters. Outperforming baseline models achieved 2.2% and 3.9% in the mean Average Precision (mAP) at Intersection over Union (IoU) 0.5 (mAP@0.5). The results demonstrate excellent effectiveness and high deployability for resource-constrained industrial scenarios.

## 1. Introduction

The surface quality of fabrics is a critical determinant of product value and customer satisfaction. Fabric defects stem from yarn irregularities, equipment wear, uneven dyeing, or human error, manifesting as spots, holes, broken threads, and texture discontinuities [1]. These defects are typically small and low-contrast against complex weave structures, rendering manual inspection inefficient and unreliable on high-speed production lines.

To address these challenges, automated visual inspection (AVI) based on computer vision (CV) has emerged as a widely adopted approach. Early methodologies relied on handcrafted features and classical image processing technology, including statistical thresholding, Gabor filters, Fourier analysis, and gray-level co-occurrence matrices [2,3]. While computationally straightforward, these approaches exhibit limited generalization capability across diverse fabric patterns and varying illumination conditions.

With the rapid advancement of deep learning—particularly convolutional neural networks (CNNs)—fabric-defect detection has achieved remarkable progress. One-stage detectors, such as YOLO and a Single-Shot Multibox Detector (SSD), deliver real-time localization [4], whereas two-stage detectors, such as Faster Region-based CNN (Faster R-CNN), enhance detection accuracy via region-proposal mechanisms [5]. For textile scenarios, researchers have incorporated mechanics, multi-scale feature extraction, and domain-specific data augmentation into these architectures to enhance robustness [6,7]. Nevertheless, practical industrial deployment still confronts key obstacles, including vulnerability to noise interference, difficulty in detecting small and low-contrast defects, and constrained computational resources on edge devices.

First, fabric images are frequently corrupted by acquisition noise arising from electromagnetic interference, unstable illumination, and environmental fluctuations in production environments. This noise tends to obscure or distort critical defect-related features, thereby elevating the rates of false positives and missed detections. Existing research has demonstrated that illumination variation and sensor noise constitute major barriers to reliable inspection on textured fabric surfaces [8]. Classical denoising methods (e.g., Gaussian and median filtering) are often insufficient for textile applications, as woven fabric processes intricate high-frequency textural features; filters of this type often blur fine defect details during preprocessing [2].

Second, fabric defects typically exhibit small sizes, irregular shapes, low contrast, and orientation traits that make them highly susceptible to being lost during standard downsampling in CNN backbones. Chen et al. noted that conventional pooling operations suppress local spatial information and diminish the model’s sensitivity to small defects in fabric inspection scenarios [9,10]. In contrast, Feature Pyramid Networks (FPNs) preserve multi-scale feature representations across network layers, thereby enhancing the detection of small defective regions [11]. Meanwhile, research on small-object detection has revealed that an interpolation-based upsampling method often smooths or dilutes subtle cues while introducing background redundancy that obscures defect boundaries [12]. Collectively, these findings underscore the necessity of integrating feature-preserving mechanisms in both the encoder (downsampling) and decoder (upsampling) stages to enhance fabric-defect detection accuracy.

Third, deployment of state-of-the-art detectors in real-world textile production lines is hampered by high computation and memory overheads. Industrial systems typically operate on edge devices—such as embedded processors, Field-Programmable Gate Arrays (FPGAs), or industrial cameras—that have constrained computational efficiency. Consequently, recent research has focused on developing lightweight CNN architectures that strike a balance between detection accuracy and computational efficiency. For example, Suryarasmi et al. proposed FN-Net, which maintains competitive detection accuracy while significantly reducing model complexity, thereby enabling real-time inspection on resource-limited hardware [13]. Similarly, Zou et al. developed a lightweight network tailored for fabric-defect detection: by leveraging pruning and structural optimization, the network reduces inference latency without compromising accuracy and exhibits strong generalization on embedded platforms [14]. In parallel, Li et al. proposed an enhanced version of the YOLOv7-Tiny framework, integrating feature-refinement modules and optimized activation functions. This modified framework achieves higher precision with substantially fewer parameters, making it suitable for real-time deployment [15]. These advances collectively underscore the critical need for detectors that are both accurate and easily deployable in practical textile manufacturing scenarios.

To address the aforementioned challenges, we propose a lightweight and interference-resilient fabric-defect detector that integrates wavelet-domain feature enhancement with efficient spatial fusion. The main contributions of this work are as follows:

- **HSV-based Channel-separation filtering:** A color-space channel separation filter leverages the perceptual characteristics of HSV to decouple brightness, hue, and saturation components. Gaussian filtering is applied in a selective manner to suppress illumination and electromagnetic noise while preserving critical structural details of fabrics.
- **Wavelet-domain feature extraction:** A Discrete Wavelet Transform (DWT) module extracts hierarchical, directional cues (horizontal, vertical, and diagonal), significantly enhancing the representation capability of texture transitions and edge anomalies across multiple scales.
- **Feature-preserving inference pathway:** An enhanced SPD block replaces conventional pooling operations in the backbone to better preserve fine-grained spatial details during downsampling by reorganizing spatial information into the channel dimension (space-to-depth). For the decoder, a novel inverse-SPD channel-concatenation strategy is adopted for upsampling; it reduces interpolation-induced background redundancy and maintains the proportion of defect-related features to a greater extent.
- **Strong accuracy–efficiency trade-off:** Experiments on the TILDA and DAGM datasets demonstrate state-of-the-art detection performance with only 3.5 M parameters, enabling seamless real-time deployment on lightweight edge devices.

The remainder of this paper is structured as follows: Section 2 presents a review of related work; Section 3 elaborates on the proposed methodology; Section 4 presents the experimental setup and results; and Section 5 provides a conclusion and discusses future research directions.

## 2. Related Work on Fabric-Defect Detection

Existing visual inspection methods for textiles can be categorized into two main types: traditional approaches and deep learning–based approaches. In practice, both types need to strike a balance between efficiency, accuracy, and anti-interference robustness [3]. Traditional technologies rely on classical image processing technologies, incorporating structural, statistical, and spectral analyses. For instance, Zhu Hao [4] proposed a feature extraction scheme based on the Redundant gray-level co-occurrence matrix (R-GLCM). Specifically, the image is decomposed at multiple scales to introduce redundancy, and then gray-level co-occurrence matrix (GLCM)-based feature extraction is performed on the redundant images, generating more discriminative defect descriptors. Qin et al. [5] extracted Gabor features from multi-channel detection and template maps, calculated the residual energy, and adopted the Maximum Mean Ratio (MMR) to quantify the salience of defects against the background texture, thus enhancing detection accuracy.

Deep learning–based detectors are generally divided into one-stage models (e.g., Single-Shot Multibox Detector (SSD) and YOLO) and two-stage models (e.g., Faster R-CNN). One-stage methods simultaneously perform classification and regression in a single forward pass, enabling high speed, while two-stage methods improve detection performance via region-proposal mechanisms [6,7,9]. In textile-specific scenarios, researchers have enhanced the detection performance of small and low-contrast defects by integrating knowledge distillation, attention mechanisms, and multi-scale feature fusion. For example, Jing [16] adapted a teacher–student (TS) framework to optimize the efficiency and accuracy of the YOLOv5 model; Guo [17] integrated a Convolutional Squeeze-and-Excitation (CSE) channel attention module into the YOLOv5 backbone, which adaptively reweights channels, thereby enhancing feature representation capability and anti-interference robustness.

Related surface inspection studies offer additional insight. For instance, some studies extend the Real-Time DEtection TRansformer (RT-DETR) by incorporating a Dual-Domain Edge Enhancement Module (DDEEM) into the backbone, enabling the capture of edge features in both spatial and frequency domains. Another line of research adopts an Efficient Multi-Scale Attention (EMA) mechanism to dynamically aggregate cross-scale features while ensuring computational efficiency. Additionally, auxiliary bounding box–based loss functions—such as Inner Intersection over Union (Inner IoU)—have been developed to enhance localization precision. These strategies highlight the effectiveness of attention mechanisms, frequency-domain information, and refined loss functions in detecting small or weakly discriminative defects. However, challenges persist in balancing accuracy and efficiency under resource constraints, and ensuring robustness against electromagnetic interference and illumination noise remains unresolved.

## 3. Methodology

**Figure 1:**
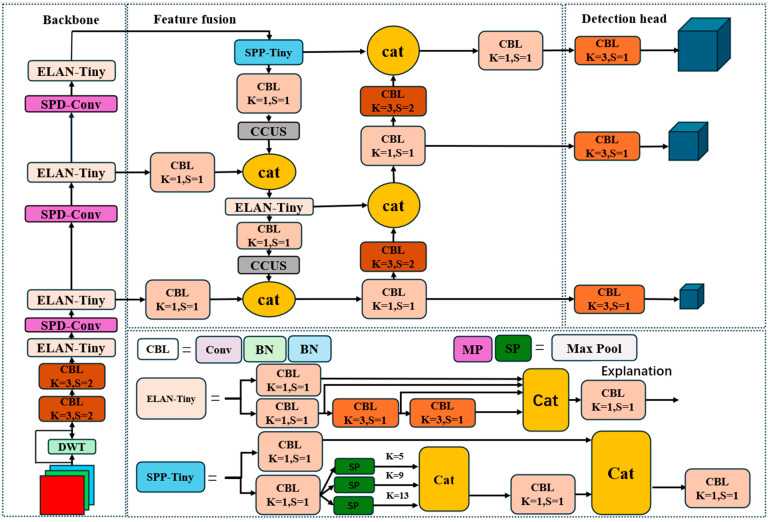
Architecture of the improved YOLOv7-Tiny–based fabric-defect detection model.

This paper presents a suite of enhancements to the YOLOv7-Tiny network. First, a wavelet transform module is integrated to strengthen the model’s capability of extracting features from complex backgrounds and defects with different shapes [18]. Second, an enhanced SPD-Conv module is employed in the backbone feature extraction network to replace the conventional pooling-based downsampling module, thereby mitigating the loss of fine-grained defect cues during downsampling [19]. Additionally, we invert and extend the SPD-Conv principle, proposing a channel-concatenation upsampling method to replace the traditional interpolation-based upsampling module in the cross-dimensional feature fusion network. This design prevents the introduction of substantial redundant background features during upsampling—features that would otherwise dilute the proportion of effective features and degrade detection accuracy. Furthermore, this paper proposes an HSV channel-separation filtering algorithm for fabric image denoising, which enhances image quality, thereby boosting the model detection accuracy [20]. The structure of the improved YOLOv7s is illustrated in Figure 1.

### 3.1. Discrete Wavelet Transform

**Figure 2:**
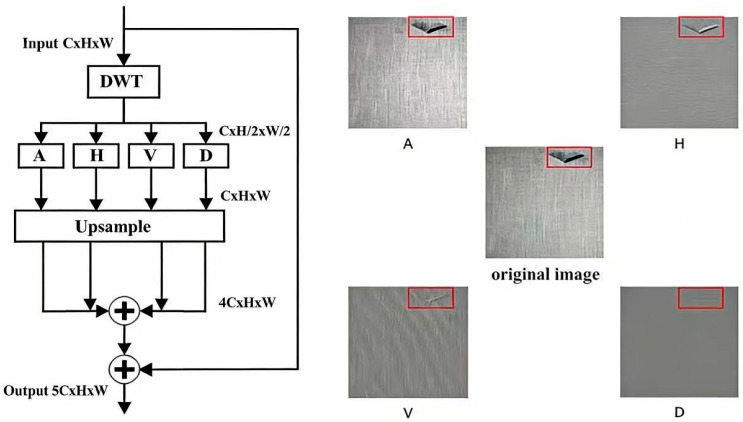
Architecture of the DWT module and its directional sub-bands (A, H, V, D).

The Discrete Wavelet Transform (DWT) module simultaneously captures spatial and frequency-domain information. Using Haar wavelets, the module alternately applies low-pass and high-pass filters along the rows and columns of the image, generating sub-bands that encode directional content—thereby enriching the feature representation of defect patterns [21]. As shown in Figure 2, the left subfigure presents the module structure, while the right subfigure illustrates the directional sub-bands.

First, we apply a one-level 2D Haar Discrete Wavelet Transform (DWT) [18] to extract directional texture cues. To keep the channel dimension compact and consistent with the “5-channel” design, the DWT is performed on the brightness component rather than on all RGB channels. Specifically, given an input RGB image $I\in R^{H\times W\times 3}$, we convert it to HSV space and take the value channel $V_{HSV}\in R^{H\times W}$. A one-level 2D Haar DWT decomposes $V_{HSV}$ into four sub-bands at half resolution, denoted as $B_{A}$ (approximation/LL), $B_{H}$ (horizontal detail/LH), $B_{V\;}$ (vertical detail/HL), and $B_{D}$ (diagonal detail/HH), each with size $\frac{H}{2}\;\times \;\frac{W}{2}$.

To align the spatial size with the original image, each sub-band is upsampled to H×W using bilinear interpolation, yielding $B_{A}^{↑},{\;B}_{H}^{↑},{\;B}_{V}^{↑},\;B_{D}^{↑}\in R^{H\times W}$. We then concatenate these maps with the original brightness channel to form a 5-channel wavelet-enhanced input:

$$X_{DWT}=Concat\;(V_{HSV},\;B_{A}^{↑},\;B_{H}^{↑},\;B_{V}^{↑},\;B_{D}^{↑})\in R^{H\times W\times 5}.$$

Insertion position. The tensor $X_{DWT}$ is used as the network input (i.e., it replaces the original 3-channel RGB input). Accordingly, the stem convolution of YOLOv7-Tiny is modified to accept 5 input channels (the rest of the backbone and head remain unchanged). This makes the integration explicit and avoids ambiguity about whether the DWT features are injected at a later backbone stage.

Computation cost. The DWT uses fixed Haar filters and introduces no learnable parameters. Its additional operations (filtering + bilinear resizing) are linear in the number of pixels and are small relative to the overall convolutional backbone, thus adding only minor overhead in practice.

On the “redundant background” concern. The “redundant background” issue discussed in this paper primarily refers to interpolation-based upsampling in the feature-fusion (decoder) stage. In contrast, the DWT branch produces frequency-oriented maps that are only resized to match H×W for concatenation; it does not synthesize new semantic background features in the decoder. Moreover, the first convolutional layers learn to fuse and reweight the five channels, which further suppresses uninformative responses.

### 3.2. SPD-Conv Downsampling Module

**Figure 3:**
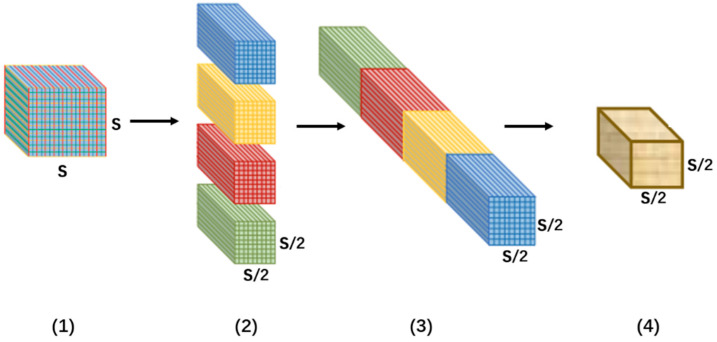
SPD module diagram. Note: The different colors (Red, Yellow, Green, and Blue) represent different feature submaps extracted from the input feature map at each sampling interval. These submaps are concatenated along the channel dimension to form the final feature map. Explanation of Numbers: (1): The input feature map, with the sampling scale S. (2): The feature map is split into submaps along the spatial dimension. (3): The submaps are concatenated along the channel dimension. (4): The final feature map, after applying a 1x1 convolution to refine the features.

The SPD module is primarily designed to mitigate the loss of fine-grained details during the downsampling process in conventional pooling. Figure 3 presents a diagram of the SPD [22] module, taking a sampling interval (scale) of 2 as an example. The core of this method lies in expanding the channel dimension by compressing the spatial dimension, thereby effectively converting spatial dimension information into channel dimension information. The specific process is as follows:

(1) $$f_{m,n}=X\left[m:S:scale,n:S:scale\right]$$

(2) $$m,n\in \left[0,scale-1\right]$$

Taking a sampling scale of 2 as an example, the specific process is as follows: First, for the input feature map, the module performs interval sampling in accordance with Formula 1, yielding four feature submaps with the dimension $C\;\times \;\frac{S}{2}\;\times \;\frac{S}{2}$. Then, these four submaps are concatenated along the channel dimension, generating a new feature map with the dimension $4C\;\times \;\frac{S}{2}\;\times \;\frac{S}{2}$. Finally, a 1 × 1 smoothing convolution is applied to refine feature representations, resulting in the final feature map (dimension: $4C\times \;\frac{S}{2}\;\times \;\frac{S}{2}$).

**Figure 4:**
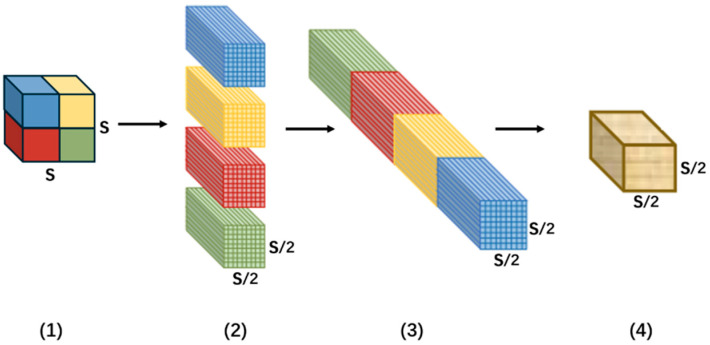
Continuous sampling for feature subgraph partitioning.

As shown in Figure 4, the SPD operation performs downscaling via a space-to-depth (pixel-unshuffled) reorganization, which preserves element information by rearranging spatial entries into the channel dimension. Note that the subsequent 1×1 convolution mixes channels and may introduce compression depending on the output channel dimension; overall, the proposed block reduces the loss of fine-grained defect cues compared with pooling-based downsampling.

(3) $$f_{m,n}=X\left[m\;\times \;\frac{S}{\mathrm{scale}}:\left(m+1\right)\;\times \;\frac{S}{\mathrm{scale}},\;n\;\times \;\frac{S}{\mathrm{scale}}:\left(n+1\right)\times \frac{S}{\mathrm{scale}}\right]$$

(4) $$m,n\in \left[0,scale-1\right]$$

Taking scale = 2 as an example. First, for the input feature map X, continuous sampling is conducted in accordance with Equations (3) and (4), yielding four feature submaps with the dimension $C\;\times \;\frac{S}{2}\;\times \;\frac{S}{2}$. Then, the four feature submaps are concatenated by channel to obtain a new feature map with shape $4C\;\times \;\frac{S}{2}\;\times \;\frac{S}{2}$. Finally, a 1 × 1 smoothing convolution is applied to smooth the features, resulting in the final feature map with dimensions of $4C\;\times \;\frac{S}{2}\;\times \;\frac{S}{2}$.

The improved SPD-Conv inherits the core benefit of space-to-depth reorganization, which is information-preserving in the sense of element rearrangement. The following 1×1 convolution performs channel mixing (and may compress features if the output channel dimension is reduced). Moreover, compared with interval sampling, the proposed continuous partition strategy maintains local neighborhood coherence to a greater extent, reducing the feature-distribution disorder and avoiding the need for additional explicit position re-mapping.

### 3.3. Channel Splicing Sampling Module

**Figure 5:**
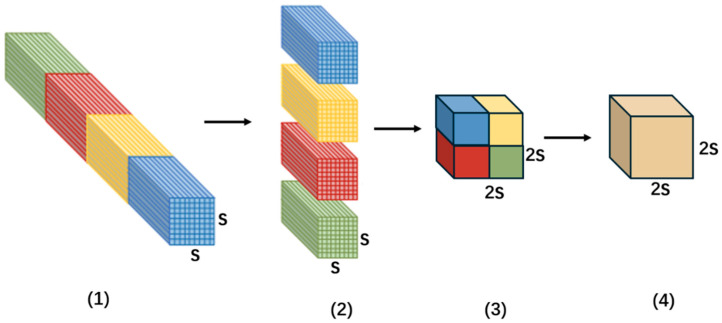
Channel splicing sampling module.

Given the small number and size of defect features, traditional interpolation-based upscaling introduces substantial redundant background information. This leads to an imbalance in the proportion of original defect features and thus degrades the model’s detection performance. To address this, we borrow and invert the core principle of SPD-Conv: using the channel dimension to directly compensate for the spatial dimension, thereby achieving upscaling without introducing any new features. The specific process is shown in Figure 5.

First, the input feature map X (dimension: C × S × S) is split along the channel dimension, yielding four feature submaps with dimension $\frac{C}{4}\;\times \;S\;\times \;S$. Then, the four feature submaps are concatenated along the spatial dimension, generating a new feature map with the dimension $\frac{C}{4}\;\times \;2S\;\times \;2S$. Finally, a 1 × 1 convolution is applied for feature smoothing, resulting in the final feature map with dimension $\frac{C}{4}\;\times \;2S\;\times \;2S$.

Compared to traditional interpolation-based upscaling, channel concatenation-based upscaling can maintain the original proportion of defect features to a greater extent. This ensures that defect features are not diluted by substantial redundant background information, thereby enhancing the model’s detection accuracy. Additionally, this module features faster internal operations than the interpolation-based method, which contributes to improving the model’s detection speed.

### 3.4. HSV Space-Based Channel-Separation Filtering Algorithm

**Figure 6:**
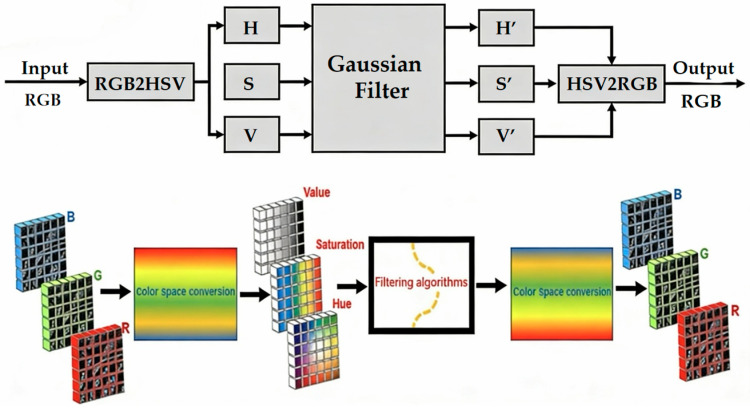
Channel-separation filtering algorithm based on HSV space (value represents the brightness channel, saturation represents the saturation channel, and hue represents the hue channel). Explanation of Colors: Red, Green, and Blue: These colors represent the individual RGB channels of the input image. The image is first converted from RGB to HSV color space. H (Hue): Represents the color information, capturing the variation in color, such as red, blue, or green. S (Saturation): Represents the intensity or purity of the color. Higher saturation means more vivid colors, while lower saturation means more grayish colors. V (Value): Represents the brightness of the color, with higher values corresponding to brighter colors. In the algorithm, each of these channels is filtered separately using Gaussian filtering to suppress noise before being recombined and converted back to RGB space for the final noise-suppressed image.

The model structure defines the performance lower bound, while dataset quality typically dictates the upper bound. Beyond the aforementioned model structure improvements, this paper further proposes a novel HSV space-based channel-separation filtering algorithm [20]. This algorithm leverages the inherent property of the HSV color space—where brightness, saturation, and hue are functionally separated—to separate the attributes of the original fabric image. Since all three RGB channels contain mixed brightness, saturation, and hue information, noise introduced by electromagnetic interference, unstable lighting, and other factors during image acquisition is barely perceptible to the naked eye. However, after attribute separation via HSV color space conversion, the noise becomes more prominent within individual HSV channels, in contrast to the RGB space. Gaussian filtering is often applied to the three HSV channels to achieve noise suppression. Finally, the denoised HSV image is converted back to the RGB space, resulting in the final noise-suppressed fabric image. The internal structure and detailed workflow of this algorithm are illustrated in Figure 6.

## 4. Experimental Results and Analysis

### 4.1. Dataset Description

**Figure 7:**
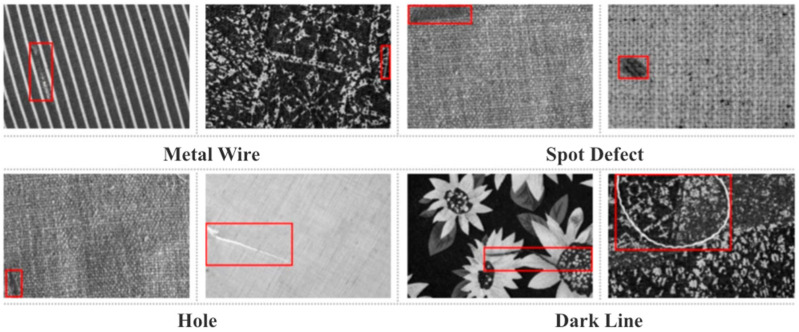
Examples from TILDA: metal wire, spot, hole, and dark line defects under different background textures.

This study employs two publicly available textile defect datasets: TILDA [23] and DAGM [21]. TILDA comprises eight fabric catalogs, including four plain weaves, two regular-patterned fabrics, and two fabrics with complex irregular patterns. For model evaluation, we focus on four typical defect categories, namely metal wire, spot, hole, and dark line. Figure 7 presents representative examples of each defect category across different background textures.

DAGM2007 is a widely used synthetic benchmark for defect detection on statistically textured surfaces. It was originally introduced for the “Weakly Supervised Learning for Industrial Optical Inspection” competition held at the DAGM 2007 [24] symposium, which was organized by DAGM (the German Chapter of the International Association for Pattern Recognition, IAPR) and the German Chapter of the European Neural Network Society (GNSS). The dataset was created by Wieler, Hahn, and Hamprecht, and it was publicly released by the Heidelberg Collaboratory for Image Processing (HCI). It contains ten texture classes (Class 1–Class 10). For each class, images are provided in 8-bit grayscale format, and defective samples are accompanied by label images in which pixel values indicate background and defective regions. In our experiments, we follow the protocol described in Section 4.1 and use the same training/testing split and evaluation pipeline as for TILDA.

**Figure 8:**
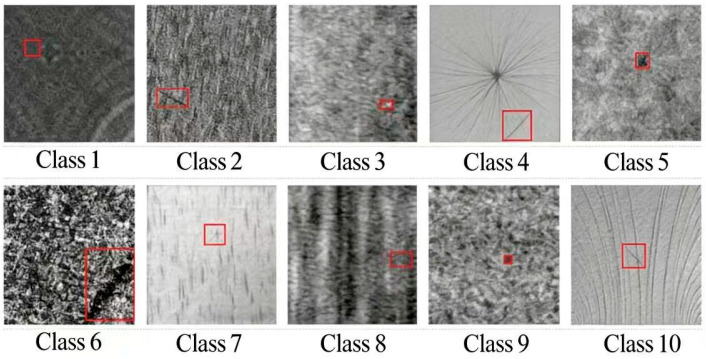
Examples from DAGM2007: metal wire, spot, hole, and dark line defects under different background textures.

We note that detection performance may vary with different train/test splits and random seeds, since they change the sample composition and optimization trajectory. To ensure fair comparison and reproducibility, we fix the split protocol for both datasets and use a fixed random seed for all experiments. The detailed settings (split strategy and seed) are reported in Section Implementation Details and Baseline Settings. Figure 8 shows representative samples of the ten DAGM2007 classes.

### 4.2. Experimental Environment

All experiments in this study were conducted on a workstation equipped with the Ubuntu Linux operating system. The hardware configuration includes an AMD EPYC 7763 64-core central processing unit (CPU) and an NVIDIA GeForce RTX 4090 graphics processing unit (GPU) with 24 GB of video memory. For model training, the number of training epochs was set to 300, the batch size was configured as 32, and the Adam optimizer was adopted for parameter updating. All other hyperparameters were kept consistent with those of the baseline YOLOv7-Tiny model; notably, binary cross-entropy (BCE) loss was employed to quantify both confidence loss and class classification loss.

#### Implementation Details and Baseline Settings

To improve reproducibility and the perceived fairness of comparisons, we explicitly summarize the training and evaluation settings for all baselines and the proposed method. Unless otherwise stated, we used the same train/test split described in Section 4.1 and adopted a fixed input resolution of 640 × 640 for both training and testing. The default training schedule was 300 epochs with a batch size of 32.

For optimization, we used the Adam optimizer. The learning-rate strategy followed a cosine decay with warm-up; the key hyperparameters (initial learning rate, weight decay, warm-up length, and other relevant settings) are reported in Table 1. To ensure comparability, we kept the core settings (data split, input resolution, total training length, and evaluation protocol) consistent across methods as much as possible. For baselines whose official training recipes differ by design (e.g., two-stage detectors vs. one-stage detectors), we followed the recommended baseline recipe while still matching the above core settings and explicitly documenting any differences in Table 2.

Data augmentation was applied consistently in terms of basic geometric and photometric transforms, including random flips, random scaling/translation, and HSV color jitter. For YOLO-based methods, Mosaic augmentation was enabled following the default configuration in the corresponding official implementations; for non-YOLO detectors, Mosaic is not directly applicable, and we therefore used equivalent geometric/photometric augmentations to maintain comparable training diversity.

Regarding implementations and initialization, YOLOv5s and YOLOv7s were run using their official repositories, while SSD/Faster R-CNN/RefineDet were implemented using a widely adopted open-source detection toolbox with standard configurations. When publicly available, models were initialized with pretrained weights (e.g., COCO pretraining) and then fine-tuned on our datasets; whether pretraining was used and whether any layers were frozen is explicitly stated in Table 1.

For evaluation, all methods used the same post-processing and metric protocol, including a fixed confidence threshold and NMS IoU threshold. Performance is reported using mAP@0.5, Precision (P), Recall (R), and F1-score (Section 4.3).

### 4.3. Evaluation Criteria

This paper evaluates detection performance using Precision (P), Recall (R), F1-score, and mean Average Precision at IoU threshold 0.5 (mAP@0.5). The corresponding calculation formulas are provided below. Note that “accuracy” is not used as an evaluation metric for object detection in this paper.

(5) $$Precision=\frac{TP}{\left(TP+FP\right)}$$

(6) $$Recall=\frac{TP}{\left(TP+FN\right)}$$

(7) $$mAP⊙0.5=\frac{1}{N}\sum_{i=1}^{N}{AP}_{i}$$

where N is the number of classes and ${AP}_{i}$ is the average precision of the class i computed from the precision–recall curve at IoU = 0.5.

(8) $$F1-Score=\frac{2\times Precision\times Recall}{\left(Precision+Recall\right)}$$

In the formulas, TP, FP, and FN denote the numbers of true positives, false positives, and false negatives, respectively. A predicted bounding box is counted as a true positive when it matches the ground-truth class and its Intersection over Union (IoU) with the ground-truth box is at least 0.5; otherwise, it is counted as a false positive. Precision and recall are computed from TP/FP/FN, and the F1-score is the harmonic mean of precision and recall.

Average Precision (AP) is defined as the area under the precision–recall (P–R) curve for each class, and mAP@0.5 is the mean of AP values over all classes at IoU = 0.5, following the PASCAL VOC-style mAP@0.5 evaluation protocol [29,30].

Additionally, to quantify model efficiency, we report computational complexity (GFLOPs) [31] and the number of trainable parameters (Params, reported in millions (M)). Note that parameter count is not the same as model size in MB, which depends on numerical precision and implementation details.

### 4.4. Results and Analysis

To evaluate the detection performance of the proposed detector, we conducted comparative experiments with representative object detectors on the TILDA and DAGM datasets. The selected baselines include SSD [25], Faster R-CNN [26], RefineDet [8], YOLOv5s [27], and YOLOv7s [28]. Note that our proposed detector is built upon YOLOv7-Tiny for lightweight deployment; YOLOv7s is included only as a heavier reference model to illustrate the accuracy–efficiency trade-off. To improve fairness and reproducibility, we explicitly report the implementation sources and training/evaluation settings for each baseline (official repositories vs. open-source toolbox configurations), and we match the core experimental conditions (data split, input resolution, total training length, and evaluation protocol) across methods as much as possible. Detailed settings are summarized in Section Implementation Details and Baseline Settings and Table 1. The results are reported in Table 2 and Table 3.

On the TILDA dataset, IR-YOLOv7-Tiny achieves mAP@0.5 = 96.8% with Precision/Recall of 94.1%/94.3% (Table 2). Compared with the YOLOv7-Tiny baseline, it improves mAP@0.5 by 0.5 percentage points while reducing parameters from 6.2 M to 3.5 M (−43.5%), indicating a stronger accuracy–efficiency trade-off for edge deployment. On the DAGM dataset, IR-YOLOv7-Tiny reaches mAP@0.5 = 98.8% with P/R = 98.73%/98.84% (Table 3), outperforming YOLOv7-Tiny by 0.2 percentage points in mAP@0.5 and 3.53 percentage points in precision, again with a substantially smaller parameter footprint. These results validate the proposed detector for practical fabric defect inspection under resource constraints.

**Figure 9:**
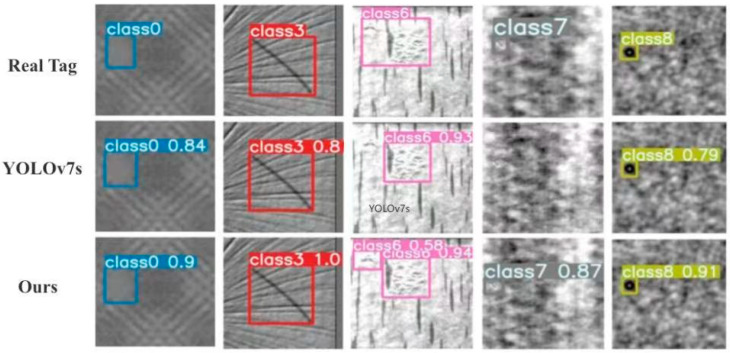
IR-YOLOv7-Tiny vs. YOLOv7s on representative samples.

To demonstrate qualitative performance gains, Figure 9 (detection effect comparison) presents representative output comparisons between IR-YOLOv7-Tiny and YOLOv7s. For the cases corresponding to columns 1, 2, and 5, IR-YOLOv7-Tiny yields noticeably more accurate detections, while for cases corresponding to columns 3 and 4, defects missed by YOLOv7s are correctly identified by IR-YOLOv7-Tiny. These results corroborate the quantitative performance improvements reported earlier.

To verify the effectiveness of the HSV channel-separation filtering strategy, we performed a corruption-based robustness evaluation, which is a common practice for assessing model generalization under non-ideal imaging conditions [32]. We synthetically corrupted fabric images with a mixture of (i) sensor noise and EMI-like stripe artifacts and (ii) global illumination perturbations that are frequently encountered in industrial vision acquisition [33,34,35]. Given an RGB image I, we applied the following degradations in the RGB space: (1) additive Gaussian noise n~N (0, σ^2^) channel-wise with σ uniformly sampled from [0.02, 0.08]; (2) periodic stripe noise modeled as s (x,y) = A·sin (2πf·t + φ) along rows or columns, where A was sampled from [0.03, 0.12], f from [2,8] cycles per image, and φ from [0, 2π], and the stripe orientation (horizontal/vertical) was chosen randomly; and (3) random global brightness scaling I ← clip (α·I) with α uniformly sampled from [0.7, 1.3].

To simulate illumination fluctuations, we perturbed the HSV-V (brightness) channel via multiplicative brightness drift and gamma change: after converting RGB to HSV, we applied $V^{\prime }=clip(\alpha \cdot V{)}^{\gamma }$, where α was sampled from [0.6, 1.4] and γ from [0.7, 1.5], and then converted the image back to RGB. All corrupted images were clipped to [0, 1]. Unless otherwise stated, both EMI-like noise and illumination perturbation were applied jointly to form the “Noisy samples” setting in Table 4. After denoising, the same detector and evaluation protocol were used to compute P/R/mAP@0.5 for Table 4, while qualitative comparisons are provided in Figure 9.

Quantitatively, joint corruption causes an absolute mAP@0.5 drop of 72.0 percentage points (96.8 → 24.8). Our CSCSF preprocessing recovers 53.1 points over the noisy input (24.8 → 77.9), retaining 80.5% of the clean-sample mAP. Moreover, CSCSF improves mAP@0.5 by 9.6 and 22.8 points compared with mean filtering and Gaussian filtering, respectively. Similar trends are observed for precision and recall, where CSCSF improves P from 39.7 to 81.1 (+41.4) and R from 30.2 to 74.4 (+44.2) relative to the noisy setting, supporting the benefit of the proposed HSV-based pre-filtering under industrial acquisition–motivated corruptions.

In the table above, after the fabric image was contaminated with noise, the model’s detection performance dropped drastically, plummeting to as low as 24.8%. Among existing classical filtering algorithms, feature compression-based filtering yielded the weakest denoising efficacy; mean filtering achieved a relatively favorable performance, restoring the model’s detection accuracy to 68.3%. The HSV color space channel-separation filtering algorithm proposed in this paper effectively leverages the functional decoupling of single channels in the HSV color space, performing targeted filtering on the brightness, hue, and saturation separately. This approach ultimately restored the model’s detection accuracy to 77.9%, significantly outperforming all comparative filtering algorithms.

In summary, the detector proposed in this paper exhibits exceptional performance across a range of key evaluation metrics, notably in parameter footprint and mAP@0.5. These results highlight its lightweight architecture and high-precision detection capability for defect inspection tasks, thus greatly improving its practical deployability in industrial scenarios.

Although our proposed detector is built upon YOLOv7-Tiny for deployability, we additionally report YOLOv7s as a heavier reference model to illustrate the accuracy–efficiency trade-off under different model scales. Therefore, YOLOv7-Tiny is treated as the primary lightweight baseline, while YOLOv7s is included only for reference comparison and is not used as the backbone for ablation.

### 4.5. Ablation Experiment

To comprehensively assess the proposed method and its constituent modules, we conducted ablation experiments on the TILDA dataset (note: corrected from “TILDA” for dataset name consistency). These experiments were designed to isolate and quantify the performance contribution of each individual module, with the detailed results presented in Table 5.

Table 5 reports ablation results based on YOLOv7-Tiny. Adding DWT, SPD, or CCUP individually improves mAP@0.5 compared with the baseline, indicating that each component contributes to defect-feature representation or feature fusion. Among single-module variants, SPD achieves the highest mAP@0.5 (97.4%), suggesting improved preservation of fine-grained defect details during downsampling.

Notably, the complete model IR-YOLOv7-Tiny is designed for deployment: it integrates all modules while adopting a lightweight configuration that reduces parameters from 6.2 M to 3.5 M (−43.5%), with only a 0.6 mAP@0.5 decrease compared with the best single-module setting. Therefore, IR-YOLOv7-Tiny achieves a favorable balance between detection accuracy and model efficiency, which is critical for edge/industrial deployment.

The experimental results in Table 5 demonstrate that each proposed component contributes to the overall performance improvement. Specifically, incorporating the DWT module enhances feature representation by injecting frequency-oriented cues, which is beneficial for capturing irregular defect textures under complex fabric backgrounds. The SPD block improves the preservation of fine-grained defect details during downsampling through space-to-depth reorganization, leading to a notable gain in detection performance. The proposed CCUP upsampling strategy mitigates interpolation-induced background redundancy during feature fusion and improves the quality of multi-scale feature aggregation.

IR-YOLOv7-Tiny reduces the parameter count from 6.2 M to 3.5 M (−43.5%) while maintaining mAP@0.5 = 96.8%, which supports efficient deployment on edge devices.

## 5. Conclusions

This paper proposes an enhanced lightweight fabric-defect detection algorithm by improving the YOLOv7s framework. It specifically addresses three core challenges in practical textile inspection: (1) high missed detection and false detection rates; (2) severe noise interference during image acquisition; and (3) poor deployability on resource-constrained devices.

To reduce electromagnetic and illumination noise inherent in industrial environments, we develop a novel color space channel-separation filtering (CSCSF) algorithm. Leveraging the functional decoupling properties of the HSV color space—brightness (V), hue (H), and saturation (S) operate independently—this algorithm applies targeted Gaussian filtering to individual channels. This design effectively suppresses noise while preserving critical defect features.

To enhance the model’s capability of capturing fine-grained defect patterns hidden in complex textures, we integrate a DWT module. This module decomposes input images into horizontal, vertical, diagonal, and global components in the spatial–frequency domain, enriching feature representation and improving robustness against background interference.

Additionally, we redesigned the network’s feature extraction and fusion modules. A modified SPD-Conv replaces traditional pooling operations in the backbone network, preserving fine-grained detail during downsampling. Furthermore, we propose a channel concatenation upsampling strategy inspired by inverted SPD-Conv, which avoids introducing redundant background information caused by interpolation and maintains the integrity of learned features during upsampling.

Experimental results on the TILDA and DAGM datasets validate the effectiveness of the proposed method. The model achieves mAP@0.5 values of 96.8% and 98.8% on the two datasets, respectively, while maintaining a compact architecture with merely 3.5 M parameters. These results confirm its potential for real-time deployment in industrial scenarios.

In future work, we plan to further explore the impact of different color spaces on noise distribution characteristics and develop adaptive noise suppression strategies for different textile materials and illumination conditions.

## Figures and Tables

**Table 1:** Summary of implementation details for baselines and the proposed method.

Model	Implementation (Codebase)	Pretrain	Input Size	Augmentation	Epochs	Optimizer	LR Schedule	Frozen Layers
SSD	Open-source toolbox config	COCO/None	640 × 640	Flip + Scale/Translate + HSV	300	Adam	Cosine + warm-up	None/[specify]
Faster R-CNN	Open-source toolbox config	COCO/None	640 × 640	Flip + Scale/Translate + HSV	300	Adam	Cosine + warm-up	None/[specify]
RefineDet	Open-source toolbox config/official impl	COCO/None	640 × 640	Flip + Scale/Translate + HSV	300	Adam	Cosine + warm-up	None/[specify]
YOLOv5s	Official repository	COCO/None	640 × 640	Flip + Scale/Translate + HSV + Mosaic	300	Adam	Cosine + warm-up	None/[specify]
YOLOv7s	Official repository	COCO/None	640 × 640	Flip + Scale/Translate + HSV + Mosaic	300	Adam	Cosine + warm-up	None/[specify]
Ours (IR-YOLOv7-Tiny)	Based on YOLOv7-Tiny (our implementation)	COCO/None	640 × 640	Flip + Scale/Translate + HSV + Mosaic	300	Adam	Cosine + warm-up	None/[specify]

Note: Mosaic augmentation follows the default setting of the official YOLO implementations. For non-YOLO detectors, Mosaic is not directly applicable; therefore, equivalent geometric and photometric augmentations (flip/scale/translation/HSV jitter) are used to provide comparable training diversity. All methods are evaluated with the same confidence threshold and NMS IoU threshold.

**Table 2:** Defect detection results on the TILDA dataset.

Model	P/%	R/%	mAP@0.5/%	F1_Score	Params (M)
SSD [25]	78.83	72.04	74.78	0.75	24.2
Faster R-CNN [26]	78.07	73.42	76.60	0.76	41.1
RefineDet [8]	74.87	92.30	82.85	0.83	43.1
YOLOv5s [27]	90.72	84.80	87.66	0.88	7.0
YOLOv7s [28]	90.90	93.80	96.30	0.92	6.2
**IR-YOLOv7-Tiny**	**94.10**	**94.30**	**96.80**	**0.94**	**3.5**

Note: Params (M) denotes the number of trainable parameters in millions. It is different from the model file size in MB, which depends on numerical precision (e.g., FP32/FP16) and serialization.

**Table 3:** Defect detection results on the DAGM dataset.

Model	P/%	R/%	mAP@0.5/%	F1_Score	Params (M)
SSD [25]	86.81	85.77	86.07	0.86	24.2
Faster R-CNN [26]	87.56	89.69	88.60	0.89	41.1
RefineDet [8]	80.09	**99.17**	95.89	0.89	43.1
YOLOv5s [27]	94.72	95.56	96.63	0.95	7.0
YOLOv7s [28]	95.20	98.10	98.60	0.97	6.2
**IR-YOLOv7-Tiny**	**98.73**	98.84	**98.80**	**0.99**	**3.5**

Note: Params (M) denotes the number of trainable parameters in millions. It is different from the model file size in MB, which depends on numerical precision (e.g., FP32/FP16) and serialization.

**Table 4:** Comparison of filter algorithms detection results.

Data processing	P/%	R/%	mAP@0.5/%
Clean sample	94.1	94.3	96.8
Noisy samples	39.7	30.2	24.8
Feature compression	39.7	30.4	24.8
Gaussian filter	65.2	53.0	55.1
Mean filtering	68.1	65.9	68.3
Color space channel separation filtering	81.1	74.4	77.9

Note: “Noisy samples” are generated by jointly adding RGB-space Gaussian + stripe noise (EMI simulation) and HSV-V brightness/gamma perturbation (illumination fluctuation), as described in Section 4.4.

**Table 5:** The ablation experiments on the TILDA dataset (based on YOLOv7-Tiny).

Model	P/%	R/%	mAP@0.5/%	F1_Score	Params (M)
YOLOv7s	90.9	93.8	96.3	0.92	6.2
YOLOv7-Tiny + DWT	95.1	95.4	97.2	0.95	6.2
YOLOv7-Tiny + SPD	94.0	95.7	97.4	0.94	6.2
YOLOv7-Tiny + CCUP	93.4	94.9	97.0	0.94	6.2
**IR-YOLOv7-Tiny**	**94.10**	**94.30**	**96.80**	**0.94**	**3.5**

Note: The ablation rows “+DWT/+SPD/+CCUP” keep the backbone width unchanged; therefore, the parameter count remains 6.2 M. The full model IR-YOLOv7-Tiny integrates all modules and adopts the lightweight design (channel/structure adjustment), reducing parameters to 3.5 M. As a result, IR-YOLOv7-Tiny provides the best accuracy–efficiency trade-off rather than the highest mAP.

## Data Availability

The data used in this study are publicly available. The TILDA fabric defect dataset can be accessed at https://universe.roboflow.com/irvin-andersen/tilda-fabric (accessed on 29 January 2026) and the DAGM 2007 competition dataset is available at https://www.kaggle.com/datasets/mhskjelvareid/dagm-2007-competition-dataset-optical-inspection (accessed on 29 January 2026).
